# Relationship of Low Vitamin B6 Status with Sarcopenia, Frailty, and Mortality: A Narrative Review

**DOI:** 10.3390/nu16010177

**Published:** 2024-01-04

**Authors:** Norihisa Kato, Akiko Kimoto, Peipei Zhang, Chanikan Bumrungkit, Sajith Karunaratne, Noriyuki Yanaka, Thanutchaporn Kumrungsee

**Affiliations:** 1 Graduate School of Integrated Sciences for Life, Hiroshima University, Higashi-Hiroshima 739-8528, Japan; m235016@hiroshima-u.ac.jp (C.B.); g230233@hiroshima-u.ac.jp (S.K.); yanaka@hiroshima-u.ac.jp (N.Y.); 2 Faculty of Health of Sciences, Hiroshima Shudo University, Hiroshima 731-3166, Japan; akimoto@shudo-u.ac.jp; 3 State Key Laboratory of Cellular Stress Biology, School of Life Science, Xiamen University, Xiamen 361102, China; zhangpeipei@xmu.edu.cn; 4 School of Medicine, Xiamen University, Xiamen 361102, China; 5 Graduate School of Innovation and Practice for Smart Society, Hiroshima University, Higashi-Hiroshima 739-8528, Japan

**Keywords:** vitamin B6, sarcopenia, skeletal muscle, imidazole peptide, satellite cells, myogenesis, frailty, mortality, aging, inflammasome

## Abstract

Marginal vitamin B6 (B6) deficiency is a widespread global concern. Inadequate B6 levels have been linked to an increased risk of age-related chronic diseases such as cardiovascular diseases and cancers. In recent years, the growing concern over sarcopenia (the age-related loss of muscle mass and strength) and frailty (a decline in physiological resilience and increased vulnerability associated with aging) is particularly relevant due to the emergence of super-aged societies in developed countries. Notably, among the thirty-one studies included in this review, twenty-five showed a significant association of B6 status with sarcopenia, frailty, and all-cause mortality in adults (*p* < 0.05), while six showed no association. Emerging studies have suggested novel mechanisms underlying this association. These mechanisms involve P2X7 receptor-mediated NLRP3 inflammasome signaling, AMPK signaling, PD-L1 signaling, and satellite cell-mediated myogenesis. Furthermore, the modulation of PLP-dependent enzymes due to B6 deficiency is associated with impaired metabolic processes, affecting energy utilization, imidazole peptide production, and hydrogen sulfide production, as well as the kynurenine pathway, all of which play vital roles in skeletal muscle health and pathophysiology. This narrative review provides an up-to-date assessment of our current understanding of the potential role of nutritional B6 status in combating sarcopenia, frailty, and mortality.

## 1. Introduction

Vitamin B6 (B6) is a water-soluble vitamin found in various foods, including cereals, fish, liver and other organ meats, potatoes and other starchy vegetables, legumes, nuts, bananas, avocados, other non-citrus fruits, egg yolks, whole grains, and vegetables. There are six isoforms of B6, also known as B6 vitamers, which include pyridoxine (PN), pyridoxal (PL), pyridoxamine (PM), and their phosphorylated forms. Among these forms, pyridoxal 5′-phosphate (PLP) is the most active and serves as a coenzyme in over 150 reactions, including the synthesis, transformation, and degradation of amino acids; the supply of one-carbon units; trans-sulfation; synthesis of tetrapyrrolic compounds, polyamines, and hydrogen sulfide (H_2_S); biosynthesis and degradation of neurotransmitters; as well as glycogen breakdown [1,2]. Additionally, B6 itself is known to have a radical scavenger activity [1].

While severe B6 deficiency is generally rare, marginal B6 deficiency (mild B6 insufficiency) appears to be common in various segments worldwide [3,4]. In fact, 5 to 25% of the male population do not receive recommended intakes of B6 in Germany, United Kingdom, and Netherland [3]. In Canadian young adult women, the prevalence of severe B6 deficiency (plasma PLP < 20 nmol/L) was 1.5% and that of marginal B6 deficiency (plasma PLP = 20–30 nmol/L) was 10.9% [4]. Furthermore, B6 deficiency poses a risk for heart diseases and cancers, making it an important vitamin in dietary considerations related to chronic diseases [1,2]. 

In recent years, the population of individuals over the age of 65 is predicted to grow worldwide. The WHO defines a super-aged society as one where the proportion of the population aged 65 and over exceeds 21% [5]. Preparations for an expected super-aged society is crucial not only from the perspectives of the medical system and healthcare, but also from socio-economic perspectives. In a super-aged society, sarcopenia (the loss of muscle mass and strength with aging) and frailty (an intermediate state between good health and a condition requiring long-term care, characterized by a decline in physiological reserve and increased vulnerability with aging) in the elderly are major concerns and have gained sufficient attention in research aimed at extending healthy life expectancy. The progression of sarcopenia or frailty is associated with the risk and prevalence of several diseases, including cardiovascular diseases, dementia, and fractures [6,7,8]. Concerning the role of nutrition in the progression of sarcopenia and frailty, epidemiological studies have highlighted the roles of nutrients such as proteins, essential amino acids, omega-3 fatty acids, and vitamin D in the context of sarcopenia, frailty, and survival [8,9]. 

Very recently, there have been noteworthy developments in understanding the relationship between B6 and age-related musculoskeletal disorders such as sarcopenia and frailty. This narrative review aims to provide an up-to-date assessment of our current understanding of the association between B6 status and sarcopenia, frailty, and mortality as well as the underlying mechanisms of the association.

## 2. Epidemiological Studies

### 2.1. Method of Literature Survey

We performed a screening of the existing literature available in the PubMed databases. Key words were used in combination with vitamin B6 with search terms such as “deficiency”, “skeletal muscle”, “sarcopenia”, “frailty”, “inflammation”, “inflammasome”, ”aging”, and “mortality”, to gather relevant information. Studies were restricted to those in the English language published between January 2006 and 30 November 2023. The search was conducted without any geographical restrictions. The following inclusion criteria were retained: human study, original study, and study of young and older adults (>18 years old, not infants, children, and pregnant or lactating). Reviews, conference proceedings, case reports, letters, summaries, expert opinions, and comments were excluded. Thirty-three original distinct studies were identified on the relationship of B6 status with sarcopenia, frailty, and mortality (Table 1, Table 2 and Table 3). Articles were considered for inclusion if they performed statistical analysis and were accessible in their full text. From the records identified through the database searching using the search terms (1960 articles), twenty-nine original distinct articles were identified on the relationship of B6 status with sarcopenia, frailty, and mortality, and are included in Table 1, Table 2 and Table 3. Additionally, four relevant articles found in the references of other articles were included in the tables (thirty-three articles in total).

### 2.2. Relatioship of B6 Status with Sarcopenia

In the general population (Table 1), all six studies [10,11,12,13,14,15] consistently demonstrated a significant inverse association of B6 nutritional status (including B6 intake, serum PLP levels, and functional B6 status (intracellular B6 status) represented by the ratio of 3-hydroxykynurenine (HK) to xanthurenic acid (XA)) and the risk of sarcopenia. Struijk et al. conducted a prospective cohort study spanning up to 3.5 years using data from the Seniors-ENRICA study, which involved Spanish older adults [10] (Table 1). Their findings indicated that B6 intake, along with increased consumption of key B6 sources, such as fish and fruit, correlated with a reduced risk of impaired mobility. 

### 2.3. Relationship of B6 Status with Frailty

Table 2 presents four studies [18,19,20,21] investigating the relationship between B6 status and frailty. In general populations, Balboa-Castillo et al. conducted a 3.5-year cohort study with 1643 community-dwelling individuals [18]. Of these, 89 (5.4%) developed frailty, with lower B6 intakes significantly associated with a higher risk of frailty. Failing to meet the recommended dietary allowances (RDAs) for B6 was also strongly linked to frailty. 

In the patients with chronic obstructive pulmonary disease (COPD), Cheng et al. [20] analyzed the data from an American population-based cross-sectional study, the National Health and Nutrition Examination Survey (NHANES) (Table 2). They observed that COPD patients with lower B6 intake had a significantly higher risk of frailty. 

### 2.4. Relationship of B6 Status with Mortality

A summary of findings from seven studies [22,23,24,25,26,27,28] examining the relationship between B6 status and all-cause mortality in the general population is presented in Table 3. Among these seven studies, six [23,24,25,26,27,28] indicated a significant inverse association between B6 status and all-cause mortality. A study by Cui et al. [22] did not indicate results for all-cause mortality, although their study indicated that B6 intake was significantly associated with a lower mortality risk from heart failure.

Zhao et al. [23] investigated the relationship between dietary B6 intake and the risk of mortality in two prospective Chinese cohorts, involving 134,480 participants. The median follow-up was 10.3 years for the Shanghai Men’s Health Study and 16.2 years for the Shanghai Women’s Health Study. Compared to the highest quintiles of B6 intake, the lowest quintiles were significantly associated with a lower risk of total (−17%), cardiovascular disease (CVD) (−27%), stroke (−29%), and coronary heart disease (CHD) (−34%) mortality in men. Women with the highest B6 intake had a 17%, 20%, and 28% lower risk of total, CVD, and stroke mortality, respectively, compared to women with the lowest B6 intake. However, no significant association was observed between dietary B6 intake and cancer mortality for among both men and women [23]. 

Concerning vascular disease-specific mortality, including CVD, CHD, heart failure, and stroke, four studies [22,23,24,26] indicated a significant inverse association with B6 status. However, regarding cancer-specific mortality, three studies [23,26,27] indicated no association between B6 status and cancer-specific mortality. Meanwhile, Bo et al. indicated a significant inverse association between B6 status and cancer-specific mortality in a general population of males, but not in a general population of women [24].

A summary of findings from fourteen studies examining the relationship between B6 status and all-cause mortality in patients with various diseases is presented in Table 3. Among the fourteen studies, nine reported a significant association [31,34,36,37,38,39,40,41,42]. Four studies [29,30,33,35] indicated no association. A study by Xu et al. [32] did not indicate results for all-cause mortality, although their study indicated that dietary B6 levels were significantly associated with reduced cancer-specific mortality.

Pusceddu et al. [39] investigated the predictive role of plasma PLP level and indices of inflammation such as interleukin-6 (IL-6) and high-sensitive C-reactive protein (hs-CRP), predicting mortality in cardiovascular patients participating in the Ludwigshafen Risk and Cardiovascular Health Study in Germany. They found that all-cause and cardiovascular-specific mortality significantly increased with higher concentrations of homocysteine and lower PLP levels. Circulating homocysteine and PLP significantly correlated with IL-6 and hs-CRP. 

Regarding disease-specific mortality, three studies [34,39,42] indicated a significant association between B6 status and CVD-specific mortality in patients. However, the relationship between B6 status and cancer-specific mortality remains inconsistent [30,32,34,35,36,38].

Summarizing the above, among the included thirty-one studies (Table 1, Table 2 and Table 3), twenty-five showed a significant association of B6 status with sarcopenia, frailty, and all-cause mortality (*p* < 0.05), whereas six showed no association (*p* > 0.05).

### 2.5. Relationship of Other Vitamin Statuses with Sarcopenia, Frailty, and Mortality

B6 status has frequently been studied alongside vitamin B12 and folate due to their interconnected role in one-carbon and homocysteine metabolisms. Among the seventeen studies including results for B12 [10,12,14,15,16,17,19,20,21,22,24,29,30,31,32,33,34] (Table 1, Table 2 and Table 3), four studies indicated a significant association with disability [14], skeletal muscle mass [17], all-cause mortality [31], and CVD-specific mortality [34]. Among the fourteen studies including results for folate [10,12,14,17,18,19,20,21,22,29,30,31,32,33], three studies indicated a significant association between the nutritional status of folate and the risks of frailty [18], heart failure [22], and all-cause mortality [31]. 

Regarding vitamin B2 [12,17,20,21,25,29,32] and A [12,14,15,16,17,19], all the studies indicated no association with sarcopenia, frailty, and mortality (Table 1, Table 2 and Table 3). Among the seven studies including results for niacin [12,17,20,25,29,31,32], only one study indicated a significant association with all-cause mortality [31]. Among the six studies including results for vitamin D [12,14,15,16,17,19], one study indicated a significant association with the loss of muscle [16]. Among the five studies including results for vitamin C [12,16,17,18,31], two studies showed a significant association with frailty [18] and all-cause and cancer-specific mortality [31]. Among the five studies including results for vitamin B1 [12,17,20,25,31], one study indicated a significant association between B1 and all-cause mortality [25]. Among the four studies [15,16,17,19] including results for vitamin E, one study indicated that frail women had significantly lower serum concentrations of α-tocopherol compared with non-frail women. Overall, few studies indicated a significant association of vitamin B12, folate, B2, A, niacin, D, C, B1, and E with sarcopenia, frailty, and mortality.

### 2.6. B6 Status and Homocysteine

Koole et al. indicated that plasma levels of homocysteine were not related to frailty [21]. A recent study by Wang et al. [34] has indicated no association between B6 status and homocysteine levels. Moreover, Grootswagers et al. reported that homocysteine concentrations did not mediate the association between B6 intake and physical performance [11].

### 2.7. Intervention Studies on the Effects of Supplemental B6 Treatment

A few studies have investigated the effects of supplemental B6 on sarcopenia, frailty, and mortality. In 2009, Ebbing et al. reported the outcomes of dietary treatment with B6 on cancer patients and all-cause mortality in two randomized controlled trials [43]. They administered B6 (40 mg/d) or placebo to patients with ischemic heart disease (n = 1705 for B6, n = 1721 for placebo) from 1998 to 2005 with follow-up until 31 December 2007. The results indicated that B6 treatment was not associated with any significant effects on cancer outcomes and all-cause mortality compared to the placebo. Other studies have explored the effects of multi-vitamin B supplements, including combinations of B6, folate, and B12, on disease outcomes and mortality [44,45]. The results have been conflicting and require further investigation.

## 3. Potential Mechanisms Underlying the Association of Low B6 Status with Sarcopenia, Frailty, and All-Cause Mortality

### 3.1. Energy Utilization

Alanine aminotransferase (ALT), a PLP enzyme, is well known to be susceptible to B6 deficiency [46]. There is accumulating evidence indicating an association of low ALT levels with increased risks of sarcopenia, frailty, and all-cause mortality [47,48,49]. There are three hypotheses to explain this association [48,49]. First, ALT is vital for converting L-alanine and α-ketoglutarate to pyruvate and glutamate in various tissues, including the skeletal muscle, heart, liver, kidney, and brain. Low ALT levels could reduce the catalytic capacity for the vital metabolic processes, such as amino acid metabolism and gluconeogenesis, leading to an increased risk of frailty and death. Second, B6 deficiency is commonly associated with low ALT levels, and B6 deficiency itself is linked to various health risks, including cardiovascular disease, immune function, depression, and neurocognitive impairment. Third, B6 is believed to enhance the activity of glycogen phosphorylase, another PLP enzyme responsible for glycogenolysis (the breakdown of muscle glycogen, a primary energy source) in skeletal muscle. Therefore, B6 supplementation could increase the utilization of muscle glycogen, potentially enhancing physical activity and improving muscle function. One study of a patient suffering from McArdle disease, a condition characterized by genetically impaired glycogen phosphorylase [50,51], suggests that B6 supplementation can improve skeletal muscle functions through enhancing residual phosphorylase activity and glycogen utilization. Further studies are required to test these hypotheses.

### 3.2. Growth of Skeletal Muscles

Sampson et al. investigated the effects of a marginal B6-deficient (1.2 mg pyridoxine (PN) HCl)/kg diet) diet on the protein fractional synthesis rate in skeletal muscle of growing rats compared to a control diet (5.8 mg PN HCl/kg diet) [52]. Their results indicated that a marginal B6-deficient diet tended to decrease the protein fractional synthesis rate (−26%, *p* = 0.053) compared to the control diet [52]. Furthermore, a study by Suidassari et al. [53] showed that a marginal B6-deficient diet (1 mg PN HCl/kg diet) significantly downregulated the gene expression of various factors that promote the growth and repair of skeletal muscle in rats, compared to the recommended dietary intake of B6 (7 mg PN HCl/kg diet). These factors include myokines, Nrf2-related factors, myogenin, and HSP60 [54,55,56], which are related to sarcopenia. Liu et al. found that supplemental B6 increased the muscle weights of fore and hind legs and the expression of myogenin and insulin-like growth factor-1 (IGF1) in growing rabbits [57]. Recently, Wang et al. reported that supplementation of B6 to a branched-chain amino acid (BCAA)-enriched formula could improve nitrogen balance and promote muscle protein synthesis through the AKT/mTOR/4EBP1 pathway in growing rats with partial gastrectomy [58]. Accordingly, dietary B6 intake may play a role in combating sarcopenia through mechanisms involving these factors and this pathway.

### 3.3. Imidazole Peptides

Studies by Suidassari et al. [59] and Kumrungsee et al. [60] revealed that compared to a low-B6 diet (1 mg PN HCl/kg diet), a diet with the recommended B6 intake (7 mg PN HCl/kg diet) significantly increased the levels of imidazole peptides, such as L-carnosine (β-alanyl-L-histidine) and L-anserine (β-alanyl-3-methylhistidine), which are ergogenic factors in skeletal and heart muscles. Notably, β-alanine, a precursor of carnosine, significantly increased, while ornithine sharply decreased. Ornithine can be metabolized to polyamines by ornithine decarboxylase (a PLP enzyme), which then can be converted to β-alanine. Therefore, B6 intake could increase the metabolism from ornithine to carnosine, which is a natually occuring histidine-containing peptide found in high concentrations in mammalian skeletal muscles and the heart.

Carnosine has various beneficial properties, including its effect on muscle contraction, and its anti-oxidant, anti-inflammatory, anti-glycation, anti-aging, pH-buffering, and glycogen phosphorylase-activating properties [61]. Of note, human blood carnosine levels in plasma and red blood cells decline in the elderly [62]. Therefore, the increased levels of carnosine resulting from supplemental B6 may be protective against aging.

### 3.4. Satellite Cells

Satellite cells in skeletal muscle may play a crucial role in maintaining skeletal muscle homeostasis and repair, while they can also be damaged by inflammation and oxidative stress [63]. A study by Komaru et al. demonstrated that a low-B6 diet led to a decrease in satellite cell pool in mouse skeletal muscle compared to a diet with an adequate level of B6 [64]. This dietary deficiency resulted in reduced proliferation and self-renewal of satellite cells during the process of myogenesis [64]. Moreover, pyridoxal (PL) has been found to increase as human myoblasts differentiate, suggesting that B6 plays an important role in myogenesis [65]. A study by Palin et al. suggested that carnosine has protective effects against oxidative damage in porcine myoblast cells [66]. This protection is believed to be mediated through the p38 MAPK intracellular signaling pathway, which is essential for cellular response to oxidative stress [63]. A subsequent study by Lie et al. has suggested that carnosine may promote muscle growth in pigs by stimulating the proliferation of satellite cells through the Akt/mTOR/S6K signaling pathway [67]. Additionally, Nagai et al. demonstrated that anserine treatment increases muscle differentiation and muscle contractility in human skeletal muscle cells [68]. Hence, the increased levels of carnosine and anserine resulting from supplemental B6 may be beneficial for muscle health and growth through mechanisms involving satellite cell-mediated myogenesis.

### 3.5. Inflammasome and Inflammation

P2X7 receptor (P2X7R)-NLRP3 inflammasome signaling has recently emerged as a target for the treatment of muscle dystrophy [69,70]. P2X7R is a purinergic receptor that responds to ATP released during cell injury and is expressed in various cells, including immune cells and muscle satellite cells. The NLRP3 inflammasome, located downstream, promotes the processing of cytokines such as interleukin-1β (IL-1β) and contributes to the progression of diseases, including skeletal muscle disorders, which trigger oxidative stress.

Earlier studies observed skeletal myositis through NF-κB signaling-mediated mechanisms [71] and reported that B6 inhibits this signaling [72]. Furthermore, Zhang et al. found that PLP inhibited NLRP3-dependent caspase-1 processing and IL-1β production and suppressed the NLRP3 inflammasome in experiments with LPS-stimulated macrophages and LPS-treated mice [73]. Furthermore, PLP has been reported to be an antagonist of P2X7R [2]. Interestingly, NLRP3 inflammasome activation is inhibited by H_2_S, produced by a PLP enzyme called cystathionine-lyase (CSE), in macrophages and in a mouse model of inflammation [74]. 

Recent evidence suggests that several elements of the kynurenine (a tryptophan metabolite) pathway have impacts on the formation, metabolism, and function of muscle and bone [75]. Some pathway elements, including kynurenine, kynurenic acid, quinolinic acid, and tryptophan metabolites, have been associated with positive or deleterious effects on muscle formation and function, involving the pathogenesis of sarcopenia and frailty. The PLP enzymes are responsible for the formation of some of the metabolites of the kynurenine pathway. For example, the inflammation and metabolic disorders caused by blocking calcium mobilization-mediated NLRP3 inflammasome activation via G protein-coupled receptor 35 (GPR35) are ameliorated by kynurenic acid, which is produced by a PLP enzyme, kynurenine aminotransferase, from kynurenine [76]. Accordingly, it is considered that B6 prevents muscle atrophy by modulating NF-κB signaling, NLRP3 inflammasome signaling, and the kynurenine pathway. This possibility needs further investigation.

AMP-activated protein kinase (AMPK), a central regulator of multiple metabolic pathways involved in the pathophysiology of aging and age-related diseases, has emerged as an important integrator of signals controlling inflammation including the inflammasome [77]. Several AMPK-dependent pathways regulate NLRP3 inflammasome activation during aging, suggesting that the NLRP3 inflammasome could be a potential pharmacological target in age-related diseases [78]. A recent study by Shan et al. has demonstrated that exposure of cultured primary macrophages to PL increases AMPK Thr172 phosphorylation in a time- and dose-dependent manner [79]. Sarcopenia is inhibited by the regulation of the AMPK/Sirt1 pathway induced by exercise and resveratrol in aged rats [80,81]. AMPK activation may constitute a therapeutic strategy for chronic inflammatory myopathy. Taken together, B6 may play a role in protecting against musculoskeletal aging by regulating inflammation and inflammasomes.

### 3.6. PD-L1

Using a metabolite library screen of cell cultures, Yuan et al. demonstrated that PL treatment significantly suppressed expression of programmed death-ligand 1 (PD-L1), which is a ligand for programmed cell death protein 1 (PD-1), through accelerated degradation in a proteasome-dependent manner [82]. PD-L1 serves as an immunosuppressor and is highly expressed in various malignancies [82]. By blocking the PD-L1 checkpoint, it can potentially serve as a prognostic marker and a target for anti-cancer immunity. PD-L1 mRNA is widely expressed in normal peripheral tissues including the skeletal muscle, heart, placenta, lungs, thymus, spleen, kidney, and liver [83]. The expression of PD-L1 is upregulated by proinflammatory cytokines and the NLRP3 inflammasome [83]. PD-L1 is upregulated in several tissues in naturally aged mice [84]. Interestingly, a recent study by Wang et al. has found that the expression of PD-L1 plays an important role in the accumulation of senescent cells and inflammation associated with aging [85]. The elimination of PD-L1^+^ senescent cells by an immune checkpoint blockade may offer a promising strategy for anti-aging therapy [85]. PD-1 is a protein on the surface of T and B cells that regulates the immune system’s response to the cells of the human body by downregulating the immune system. Recently, Liu et al. has suggested that PD-1 plays a crucial role in regulating muscle inflammation, angiogenesis, blood perfusion, and overall exercise capacity in mice after hindlimb ischemia [86]. Taken together, B6 may have a role in anti-inflammation and anti-aging by suppressing PD-L1. Further study is needed to test this hypothesis.

### 3.7. Musculoskeletal Aging with Low B6 Status and Other Age-Related Disorders

There is evidence that sarcopenia and frailty are significantly associated with impaired cognitive function and fractures [6,7]. Recent studies have shown an association of low B6 status with brain function and bone loss, although these studies are limited and remain to be fully explored [87,88]. Thus, a question is raised as to whether the impaired cognitive function and bone metabolism caused by low B6 status lead to sarcopenia and frailty. Sarcopenia and frailty are common in patients with heart failure and are strongly associated with the prognosis [89]. Supplemental B6 plays an important role in cardioprotection [2]. Accordingly, it is necessary to investigate the possibility that the protective effects of B6 on the brain, bone, and heart lead to reduced risks of sarcopenia and frailty.

## 4. Outlook for Future Research

Currently, intervention studies on the effects of supplemental B6 on the risk of sarcopenia, frailty, and mortality are very limited. In understanding the role of B6 status in healthy aging of older adults, intervention studies must be conducted to investigate the effects of supplementing B6 in a marginal B6-deficient diet on sarcopenia, frailty, and mortality in the elderly. Previous studies on the relationship of B6 status with disease-specific mortality, particularly cancer-specific mortality, are limited and conflicting, although there is increasing evidence indicating an association between B6 status and all-cause mortality. Thus, further study is necessary to investigate the relationship of B6 status with cancer-specific mortality. In terms of mechanistic studies on the association with B6 status, clinical studies are still limited, although studies with animal models and cell cultures are accumulating. Thus, further mechanistic clinical studies are necessary.

Sarcopenia is often accompanied by an increase in fat mass, and this condition is defined as sarcopenic obesity [90]. The prevalence of sarcopenic obesity is increasing worldwide, especially in the aging population. Surprisingly, a recent meta-analysis by Eitmann et al. demonstrated that the age-dependent impact of additional obesity on all-cause mortality in sarcopenic people results in lower mortality risk in older adults, i.e., the obesity paradox [91]. It has been reported that circulating B6 levels were negatively related to obesity in middle-aged and older adults [92,93]. Additionally, B6 status in obese adults was positively associated with health consequences [17,34]. Accordingly, it is of great interest to investigate the impact of B6 status on sarcopenic obesity and sarcopenic non-obesity and the associated consequences.

## 5. Conclusions

**Figure 1:**
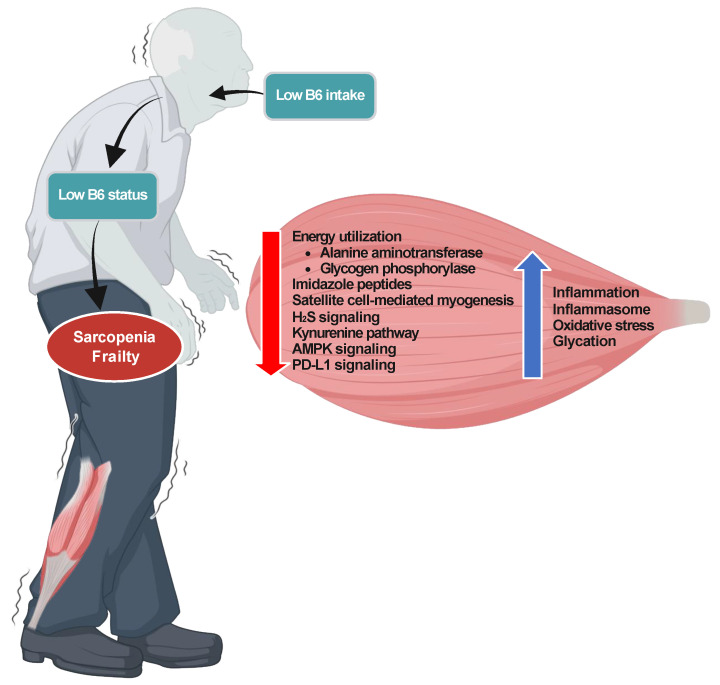
Potential underlying mechanisms linking low B6 status to sarcopenia and frailty. Created with BioRender.com (accessed on 5 December 2023).

In this review, available evidence of the relationship of nutritional B6 status with sarcopenia, frailty, and mortality was summarized. Among the thirty-one studies included in this review, twenty-five showed a significant association of B6 status with sarcopenia, frailty, and all-cause mortality in adults. Conversely, few studies indicated a significant association with other vitamins such as vitamins A, B1, B2, niacin, folate, B12, D, C, and E. These studies highlight the significance of B6 status in relation to musculoskeletal aging. Furthermore, emerging experimental evidence underlying the association suggests the potential effects of B6 on musculoskeletal aging through modulations of PLP enzymes, imidazole peptides, NF-κB signaling, NLRP3 inflammasome signaling, the kynurenine pathway, AMPK signaling, the PD-L1 signaling pathway, and satellite cell-mediated myogenesis (Figure 1). Available evidence from epidemiological and mechanistic studies indicates that B6 plays a potential role in combating sarcopenia, frailty, and all-cause mortality as an anti-aging factor. This review provides an insight into the impact of supplemental B6 against musculoskeletal aging. However, intervention studies on the effects of supplementing B6 in a marginal B6-deficient diet on sarcopenia, frailty, and mortality in animal models of aging and older adults are necessary.

## Figures and Tables

**Table 1:** Summarized findings of the studies assessing the relationship between B6 status and sarcopenia in general adult population and patients.

References, Publication Year (Recruiting Area)	Assessment of B6 Status	Study Design	Study Population	Key Findings
***General population***				
Struijk et al. [10] 2018 (Spain)	B6 intake	Cohort (median follow-up of 3.5 years)	n = 1630 participants (≥60 years, male 51–53%)	**(+)** The OR for highest vs. lowest tertile of **B6** intake was 0.66 (95% CI, 0.44–0.99; *p*-trend < 0.05). No association was found between B12 and folate intakes and physical function.
Grootswagers et al. 2021 [11] (Italy, UK, Netherland, Poland, France)	B6 intake	Cross-sectional	1249 healthy older adults (65–79 years, male, 36–50%)	**(+)** Positive associations between **B6** intake and chair rise test performance in the full population (*p* < 0.05) and between **B6** intake and handgrip strength in participants with low physical activity were observed (*p* < 0.05). Homocysteine concentrations did not mediate these associations.
Park et al. [12] 2022 (Korea)	B6 intake	Cohort (up to 10 years)	801 participants (70–84 years, 48% male)	**(+)** The highest quartile of **B6** intake was 0.45-fold lower for the risk of sarcopenia compared to the lowest quartile (HR = 0.45; 95% CI: 0.22–0.91; *p*-trend = 0.025). Intakes of retinol, D, K, B1, B2, niacin, C, B12, and folate were not associated with sarcopenia.
Magalhães et al. [13] 2023 (Brasil)	B6 intake	Cross-sectional	295 community-living older adults (>60 years, mean age 70 years, male 18%)	**(+)** Seventy eight % of the participants had insufficient intake of **B6**. Inadequancy of B6 (OR = 2.18; 95% CI: 1.03–4.64) was identified as risk factor for sarcopenia (*p* < 0.05).
Bartali et al. [14] 2006 (USA)	Serum PLP	Cohort (up to 3 years)	643 women (≥65 years)	**(+)** Women in the lowest quartile of serum concentrations of **PLP** (HR = 1.31; 95% CI: 1.03–1.67), B12 (1.40; 1.12–1.74), and Se (1.38; 1.12–1.71) had higher risk of disability compared with women in the upper 3 quartiles (*p* < 0.05). Serum levels of retinol, 25(OH)D, folate, and Zn were not associated with disability.
Lu et al. [15] 2020 (Singapore)	Serum HK/XA ratio	Cross-sectional	189 participants (mean age 73.2 years, male 37%)	**(+)** Compared with non-sarcopenic adults, sarcopenic adults had higher **HK/XA ratio** (OR = 1.198; 95% CI: 1.010–1.420; *p* = 0.038), indicating a deficiency of **B6**. Serum levels of retinol, 25(OH)D3, α-tocopherol, and total B12 did not differ.
***Patients***				
Takahashi et al. [16] 2021 (Japan)	B6 intake	Cohort (mean 13.7-month follow-up)	197 patients with T2DM (mean age 72 years, 57% male)	**(−)** Intake of D was significantly related to the loss of muscle mass (OR = 0.93; 95% CI: 0.88–0.97; *p* = 0.003). However, intakes of A, B2, **B6**, B12, C, and E were not related.
Lim et al. [17] 2023 (Korea)	B6 intake	Cross-sectional	59 women (19–65 years, BMI values of 25–32 kg/m^2^)	**(+)** Intakes of **B6** and B12 intake (**B6**: β = 0.338, *p* = 0.012; B12: β = 0.281, *p* = 0.024) showed positive associations with the skeletal muscle mass-to-visceral fat area ratio. Intakes of other vitamins such as A, D, E, K, B1, B2, niacin, panthothenic acid, C, and folate showed no association.

OR = odds ratio; HR = hazard ratio; CI = confidence interval; B6 = vitamin B6; PLP = pyridoxal 5′-phosphate; B1 = vitamin B1; B2 = vitamin B2, B12 = vitamin B12, C = vitamin C; A = vitamin A; K = vitamin K; HK/XA ratio = 3-hydroxykynurenine/xanthurenic acid ratio (index for intracellular B6 status); D = vitamin D; 25(OH)D = 25-hydroxyvitamin D; T2DM = type 2 diabetic mellitus; E = vitamin E; (+) significant and (−) non-significant association between B6 status and sarcopenia. List of abbreviations is shown below.

**Table 2:** Summarized findings of the studies assessing the relationship between B6 status and frailty in general adult population and patients.

References, Publication Year (Recruiting Area)	Assessment of B6 Status	Study Design	Study Population	Key Findings
***General population***				
Balboa-Castillo et al. [18] 2018 (Spain)	B6 intake	Cohort (up to 3.5 years)	1643 community-dwelling individuals (≥65 years, 50% male)	**(+)** The ORs (95% Cl) of frailty for those in the lowest versus the highest tertile of vitamin intake were 2.80 (1.38–5.67), *p*-trend 0.004, for **B6**; 1.65 (0.93–2.95), *p*-trend 0.007, for C; 1.93 (0.99–3.83), *p*-trend 0.06, for E; and 2.34 (1.21–4.52), *p*-trend 0.01, for folates. Non-adherence to the RDAs of **B6** was related to frailty (OR 2.23; 95% CI: 1.30–3.83; *p* < 0.01).
Semba et al. [19] 2006 (USA)	Serum PLP	Cohort (up to 3 years)	766 female participants (≥65 years)	**(−)** Compared with women in the upper three quartiles, women in the lowest quartile of serum carotenoids (HR = 1.39; 95% CI: 1.01–1.92) and α-tocopherol (HR = 1.39; 95% CI: 1.02–1.92) had an increased risk of becoming frail (*p* = 0.04). Other nutrients such as retinol, **PLP**, B12, and folate were not significantly associated with the incidence of frailty.
***Patients***				
Cheng et al. [20] 2023 (USA)	B6 intake	Population-based cross-sectional	1201 COPD patients (mean age 63.9 years, 62% male)	**(+)** The association between **B6** intake and frailty risk (OR = 0.80; 95% CI: 0.66–0.95; *p* = 0.013) was significant. Intakes of B1, B2, niacin, folate, and B12 were not associated with frailty risk.
Koole et al. [21] 2021 (Netherland, Germany)	Serum PLP	Cohort (up to 6 months)	1676 diagnosed stage I–III colorectal cancer patients (mean age 65.6 years, 64% male)	**(+) B6** status was found to be associated with better physical functioning, better social functioning, and less fatigue (*p* < 0.05). Dose–response relationships were found between **B6** status and quality of life outcomes. Plasma levels of B2, folate, cobalamin, and homocysteine were not related to frailty.

COPD = chronic obstructive pulmonary disease; RDA = recommended dietary allowance; (+) significant and (−) non-significant association between B6 status and frailty.

**Table 3:** Summarized findings of the relation between B6 status and mortality in general adult population and patients.

References, Publication Year (Recruiting Area)	Assessment of B6 Status	Study Design	Study Population	Key Findings
***General population***				
Cui et al. [22] 2010 (Japan)	B6 intake	Cohort (median 14-year follow up)	58,730 participants (40–79 years, 39% male)	The HRs (95% CI) of the mortality from heart failure for the highest versus lowest quintiles were 0.39 (0.15–1.00) for **B6** and 0.50 (0.27–0.94) for folate intakes in men (*p*-trend < 0.05), and the HR of coronary heart disease (CHD) was 0.57 (0.34–0.96) for folate intakes in women (*p*-trend < 0.05). No association was found between B12 intake and mortality from CHD.
Zhao et al. [23] 2019 (China)	B6 intake	Cohort (median 10.3 year follow-up for men and median 16.2-year follow-up for women)	134,480 participants (40–74 years, 44% male)	**(+)** The HR for the highest vs. lowest quintiles of **B6** for total, cardiovascular disease (CVD), stroke, and CHD mortality among men was 0.66–0.83 (*p*-trend < 0.01). Similar association was found in women. No significant association was observed between dietary **B6** and cancer mortality among both men and women.
Bo et al. [24] 2022 (USA)	B6 intake	Two cohort (median 9.8-year follow-up)	55,569 participants (mean age 49 years, 52% male)	**(+)** In men, the HRs (95% CI) for the highest versus lowest quintiles of folate and **B6** were 0.77 (0.71–0.85) and 0.79 (0.71–0.86) for all-cause mortality, 0.59 (0.48–0.72) and 0.69 (0.56–0.85) for CVD mortality, and 0.68 (0.56–0.84) and 0.73 (0.60–0.90) for cancer mortality, respectively (*p* trend < 0.05). Among women, the HRs (95% CI) for the highest versus lowest quintiles of folate and **B6** were 0.86 (0.78–0.95) and 0.88 (0.80–0.97) for all-cause mortality and 0.53 (0.41–0.69) and 0.56 (0.44–0.73) for CVD mortality, respectively (*p* trend < 0.05). No significant associations between dietary B12 and all-cause and cause-specific mortality were observed.
Huang et al. [25] 2012 (Taiwan)	Plasma PLP	Cohort (up to 8 years)	1747 participants (≥65 years, 50% male)	**(+)** Relative to the lowest tertile of B1 or **B6** intakes, the HRs (95% CI) for 3rd tertile were 0.74 (0.58–0.95) and 0.74 (0.57–0.97) (both *p*-trend < 0.05) for all-cause mortality, respectively. No significant associations were observed between niacin and B2 and all-cause mortality.
Yang et al. [26] 2021 (USA)	Serum PLP	Cohort (up to 5 years)	12,190 participants (mean age 46.6 years, 49% male)	**(+)** The participants with higher serum **PLP** had lower risk of all-cause mortality (HR = 0.85) and lower risk of CVD mortality (HR = 0.81) for each unit increment in natural log-transformed **PLP**, respectively. A higher log-transformed **PLP** was not significantly associated with a lower risk for cancer-specific mortality. Compared with sufficient **B6**, deficient (HR = 1.37) and insufficient (HR = 1.19) **B6** levels were significantly associated with a higher risk for all-cause mortality.
Schorgg et al. [27] 2022 (USA)	Serum PA/PLP ratio	Cohort (median 7.8-year follow-up)	15,304 participants (20–85 years, 48% male)	**(+)** A positive association between the **PA/PLP ratio** and all-cause mortality was observed with the HR (5th vs. 1st quitile) of 1.45 (95% CI: 1.14–1.85; *p*-trend < 0.0001). There were no significant associations of **B6** turnover with CVD or cancer-specific mortality.
Dugué et al. [28] 2022 (Australia)	Serum HK/XA ratio and PAr index	Cohort (median 14.3-year follow-up	41,513 participants (27–88 years, 48% male)	**(+)** Associations with all-cause mortality were observed for several markers of **B6**-related factors, including IL-6, neopterin, quinolinic acid, kynurenine/tryptophan ratio, cystatin C, **HK/XA**, calprotectin, **PAr index**, serum amyloid A, TNF-α, anthranilic acid, 3-hydroxykynurenine, 3-hydroxyanthranilic acid, and 4-pyridoxic acid (*p* < 0.05).
***Patients***				
Xu et al. [29] 2008 (USA)	B6 intake	Cohort (mean 5.6-year follow-up)	1508 women with breast cancer (mean age 59 years)	**(−)** Higher intakes of B1 were associated with reduced all-cause mortality (HR = 0.44; 95% CI: 0.24–0.81; *p*-trend = 0.01). However, intakes of **B6**, folate, B2, niacin, and B12 were not associated with all-cause mortality.
Lochhead et al. [30] 2015 (USA)	B6 intake	Cohort (median 14.9-year follow-up)	1550 patients with stage I–III colorectal cancer (mean age 66 years, 31% male)	**(−)** No significant associations were observed between postdiagnostic intakes of folate, **B6**, B12, and methionine and colorectal cancer-specific and all-cause mortality (*p*-trend ≥ 0.13).
Ricci et al. [31] 2020 (South Africa, Germany, France)	B6 intake	Cohort (median 5.7-year follow-up)	2371 cancer survivors (≥19 years, 42.4% male)	**(+)** The highest versus the lowest tertiles of intakes of **B6**, B1, folate, B12, niacin, and C were inversely associated with all-cause mortality (HR = 0.55–0.75; *p*-trend < 0.05). Intakes of **B6**, B1, B12, niacin, and C were inversely associated with cancer-specific mortality (HR = 0.39–0.63; *p*-trend < 0.05).
Xu et al. [32] 2022 (China)	B6 intake	Cohort (median 37.2-month follow-up)	635 newly diagnosed ovarian cancer patients (18–79 years)	A reduced ovarian cancer-specific mortality with the highest compared with the lowest tertile of dietary **B6** (HR = 0.52, 95% CI: 0.32–0.84, *p*-trend < 0.05) was found. No significant associations with ovarian cancer mortality were observed for intakes of B2, niacin, folate, and B12. A curvilinear association between **B6** intake and ovarian cancer mortality was found (*p* for non-linearity < 0.05).
He et al. [33] 2022 (China)	B6 intake	Cohort (median 791-day follow-up)	905 diagnosed hepatocellular carcinoma patients (mean 51.9 years)	**(−)** There was no significant association of intakes of B6, folate, B12, B2, or niacin with all-cause mortality and with hepatocellular carcinoma-specific mortality (*p* for non-linearity > 0.05).
Wang et al. [34] 2023 (USA)	B6 intake	Cohort (median 15.7-year follow-up)	7718 obesity adults (mean age 50 years, 20–85 years, 37% male)	**(+)** Folate intake was independently associated with a decreased incidence of all-cause mortality (HR = 0.71; 95% CI: 0.58–0.87; *p*-trend < 0.01). Higher intakes of **B6** and B12 were inversely correlated with CVD mortality (0.63, 0.40–0.98 and 0.44, 0.29–0.65, respectively, all *p*-trends < 0.001), but not associated with cancer mortality. **B6** intake was not associated with homocysteine levels.
Je et al. [35] 2013 (Korea)	Plasma PLP	Cohort (up to 7 years)	472 patients with colorectal cancer (40–84 years, 68% male)	**(−)** Compared with patients who had less than 45 nmol/L of plasma **PLP**, those who had 110 nmol/L had HRs of 0.85 (95% CI: 0.50–1.45) and 0.87 (0.56–1.35) for colorectal cancer-specific and overall mortality (*p*-trend > 0.2).
Muller et al. [36] 2015 (Czech)	Plasma PLP	Case cohort (median 2.6-year follow-up)	630 renal cell carcinoma patients (62% male, ≥18 years)	**(+)** The HR of the cancer-speficic death mortality for renal cell carcinoma patients was significantly lower among those in the highest compared to the lowest fourth of **PLP** concentration (HR (4th vs. 1st quartile) = 0.33; 95% CI: 0.18–0.60; *p*-trend < 0.001).
Ulvik et al. [37] 2016 (USA)	Plasma PLP, Par	Cohort (up to 8.8 years)	3749 patients with acute myocardial infarction (mean age 63 years, 73% male)	**(+) PAr**, a proposed marker of **B6** catabolism, predicted all-cause mortality. **PAr** provided an HR per SD of 1.31 (95% CI: 1.21–1.41) in patients hospitalized for acute myocardial infarction in a significant manner.
Minovic et al. [38] 2017 (Netherlands)	Plasma PLP, HK:XA ratio (a marker of functional B6 status)	Cohort (median 5.3-year follow-up)	678 renal transplant recipients (RTRs) and 297 health control (mean age 54 years for control, and 53 years for RTRs, 55% male)	**(+)** RTRs had a higher median **HK/XA** than healthy controls (*p* < 0.05). In RTRs, the **HK/XA** was inversely associated with plasma **PLP** (*p* < 0.001). A higher **HK/XA** was significantly associated with increased risk of all-cause mortality, cancer mortality, and infectious disease mortality in RTRs (*p* < 0.05), but not with CV mortality (*p* > 0.05).
Pusceddu et al. [39] 2020 (Germany)	Plasma PLP	Cohort (median 9.9-year follow-up)	2968 patients with coronary artery disease (48–76 years, 70% male)	**(+)** Patients in the 4th quartile of **PLP** had an HR for all-cause mortality of 0.41 (95% CI: 0.33–0.49) and for CVD mortality of 0.40 (0.33–0.49), compared to those in the 1st quartile. All-cause and CVD mortality significantly increased with higher concentrations of homocysteine and lower **PLP**.
Zhang et al. [40] 2022 (China)	Serum PLP	Cohort (median 85-month follow-up)	2574 patients with T2DM (mean age 62.3 years, 51% male)	**(+)** The HRs and 95% CIs from lowest to highest serum levels of **PLP** (<21.4, 21.4–35.8, 35.7–63.6, and >63.6 nmol/L) were 1.00 (reference), 0.93 (0.71–1.23), 0.85 (0.64–1.13), and 0.74 (0.55–0.99), respectively, for all-cause mortality (*p*-trend = 0.035).
Holowatyj et al. [41] 2022 (Germany, USA, Netherland, Austria)	Serum PLP	Cohort (median 3.2-year follow-up)	2031 patients diagnosed with stage I–III colorectal cancer (>18 years, 64% male)	**(+)** After a median follow-up of 3.2 years for overall survival, higher preoperative **B6** status was associated with 16–32% higher all-cause and disease-free survival, although there was no significant association with disease recurrence.
Cui et al. [42] 2023 (China)	Serum PLP	Cohort (mean 11-year follow-up)	5434 participants with hypertension (mean age 58.5 years, 50% male)	**(+) PLP** was negatively associated with CVD mortality (HR [95% CI] 4th vs. 1st quartile: 0.66; 0.47–0.94; *p*-trend = 0.03). Similarly, a higher quartile of **PLP** was associated with a lower risk of all-cause mortality (0.67; 0.56–0.80; *p*-trend < 0.01).

CHD = coronary heart disease; CVD = cardiovascular disease; PA/PLP ratio = pyridoxic acid/PLP ratio (a marker of B6 turnover); PAr index = PA/(PL + PLP) (a marker of B6 catabolism); SD = standard deviation; IL-6 = interleukin-6; RTR = renal transplant recipients; (+) significant and (−) non-significant association between B6 status and all-cause mortality. The studies of references [22,32] did not show results for all-cause mortality.

## Abbreviations

B6	vitamin B6
PLP	pyridoxal 5′-phosphate
PN	pyridoxine
PL	pyridoxal
PA	pyridoxic acid
HK	3-hydroxykynurenine
XA	xanthurenic acid
OR	odds ratio
HR	hazard ratio
CI	confidence interval
25(OH)D	25-hydroxyvitamin D
T2DM	type 2 diabetic mellitus
COPD	chronic obstructive pulmonary disease
B1	vitamin B1
B2	vitamin B2
B12	vitamin B12
CVD	cardiovascular disease
CHD	coronary heart disease
NHANES	National Health and Nutrition Examination Survey
PAr index	(pyridoxic acid)/(pyridoxal + pyridoxal 5′-phosphate) index
SD	standard deviation
IL-6	interleukin-6
hs-CRP	high sensitive C-reactive protein
RTR	renal transplant recipient
A	vitamin A
C	vitamin C
E	vitamin E
RDA	recommended dietary allowance
ALT	alanine aminotransferase
BCAA	branched chain amino acids
IGF1	insulin-like growth factor-1
Nrf2	NF-E2-related factor 2
HSP	heat shock protein
AKT	Akt serine/threonine kinase (also called protein kinase B)
mTOR	mammalian target of rapamycin
4EBP1	eukaryotic initiation factor 4E binding protein 1
S6K	p70 S6K kinase
L-carnosine	β-alanyl-L-histidine
L-anserine	β-alanyl-3-methylhistidine
MAPK	mitogen-activated protein kinase
NLRP3	nucleotide-binding oligomerization domain-like receptor family, pyrin domain-containing 3
NF-κB	nuclear factor-kappa B
LPS	lipopolysaccharide
AMPK	AMP-activated protein kinase
Sirt1	NAD^+^-dependent histone deacetylase (also called Sirtuin 1)
PD-L1	programmed death-ligand 1
PD-1	programmed cell death protein 1
